# Virtual consultations for oral surgery patients

**DOI:** 10.1186/s12903-022-02076-7

**Published:** 2022-03-21

**Authors:** Aoife Crummey, Abigail Graham, Eleni Besi

**Affiliations:** 1 Dental Core Trainee, Oral Surgery Department, Edinburgh Dental Institute, Lauriston Building, 39 Lauriston Place, Edinburgh, EH3 9HA UK; 2 Oral Surgery Consultant, Oral Surgery Department, Edinburgh Dental Institute, Lauriston Building, 39 Lauriston Place, Edinburgh, EH3 9HA UK

**Keywords:** Virtual consultation, Teledentistry, Digital communication, Patient information leaflets, Informed consent

## Abstract

**Background:**

Following Covid-19, many departments have incorporated teledentistry into practice. As new consultation methods are introduced, it’s imperative that patients have as similar an experience with virtual consultations to ensure informed decision-making. This project evaluated patients' perceptions of video consultations and determined if patients seen virtually received the same standard of information by auditing compliance with sending patient information leaflets (PILs) following video consultation.

**Method:**

The department’s PILs were used to create an inclusion list for patients requiring a PIL. A retrospective audit assessed the notes of 100 video consultations for records of if PILs were sent and by what method. The department’s PILs were digitalised and a clinical mailbox introduced enabling clinicians to email patients a PIL hyperlink. The audit was repeated for 88 video consultations. Patient and staff feedback was gathered via online surveys.

**Results:**

Initially, 51% of cases met the criteria requiring a PIL and 16% of patients were sent PILs. Following mailbox introduction, 53% of cases met the criteria and 94% were sent PILs, 100% via email. Patient and staff feedback was positive regarding video consultations and digital PILS. Technical difficulties were reported in 44% of cases.

**Conclusions:**

Patients perceive virtual consultations to be a positive change and the introduction of a mailbox enhances video consultations in an efficient and cost-effective manner. Patient information can be standardised, via digital PILs, regardless of consultation type. As departments implement post-pandemic changes, utilisation of a mailbox can provide multiple improvements to care.

## Background

The World Health Organisation defines telemedicine as “the delivery of healthcare services, where distance is a critical factor, by all healthcare professionals using information and communication technologies for the exchange of valid information for diagnosis, treatment and prevention of disease and injuries, research and evaluation, and for the continuing education of health care providers, all in the interests of advancing the health of individuals and their communities” [1].Telemedicine has previously been used in the dental field, teledentistry, to increase access to specialist care and screening for remote and rural populations and to support local delivery of treatment [2, 3]. Teledentistry is defined as “the practice of using videoconferencing technologies to diagnose and provide advice about treatment over a distance" [4]. It can be synchronous, live communication involving a real time discussion between patient and clinician or asynchronous, where information such as history and clinical images are collated and sent to a clinician for diagnosis and treatment, avoiding the need for live interaction.

Since the beginning of the Covid-19 pandemic and the initial suspension of all routine dental provision [5], teledentistry has gained significant popularity as an alternative to face-to-face consultations [6–10]. The introduction of virtual clinics enabled patient consultations to take place whilst following guidance to limit hospital footfall and maintain social distancing [11]. In many departments, teledentistry has been incorporated into standard practice and the remobilisation of services to reducing waiting lists [12]. Regulatory bodies have issued guidance for clinicians regarding teleconsultations and recommend that patients receive the same standard of care as that of face-to-face-healthcare [13]. The General Dental Council advise that remote consultations can be more convenient for patients and a beneficial use of resources but note that patients need to be given information about all the options available to them, including declining treatment, in a way they can understand [14].

Prior to the COVID-19 pandemic, all patients referred to the Oral Surgery Department at the Edinburgh Dental Institute (EDI) had an initial in person consultation and if appropriate were given a paper patient information leaflet (PIL) about their condition or the planned procedure. This ensured all patients had sufficient standardised information following their appointment and provided a reference for if they wished to consider this information further. Following changes in practice due the pandemic, many patients have an initial synchronous virtual consultation through a video link using “Near Me” on the Attend Anywhere Platform. The majority of clinicians connect to these consultations from an administration area within the department but the platform allows some clinicians to connect from home using a secure remote connection to access patient records. This project was undertaken to evaluate patients’ and clinicians’ satisfaction of virtual clinics and if patients seen virtually were receiving the same standard of reference information as they would have pre-pandemic, or if they were seen on clinic in person.

### Implementation of clinical mailbox and digital PILs

Following the first cycle of this project, the department’s PILs were updated and digitalised. A generic clinical email mailbox was set up which all department members could access and send emails from. An email template was designed containing a hyperlink to each digital PIL.

**Fig. 1:**
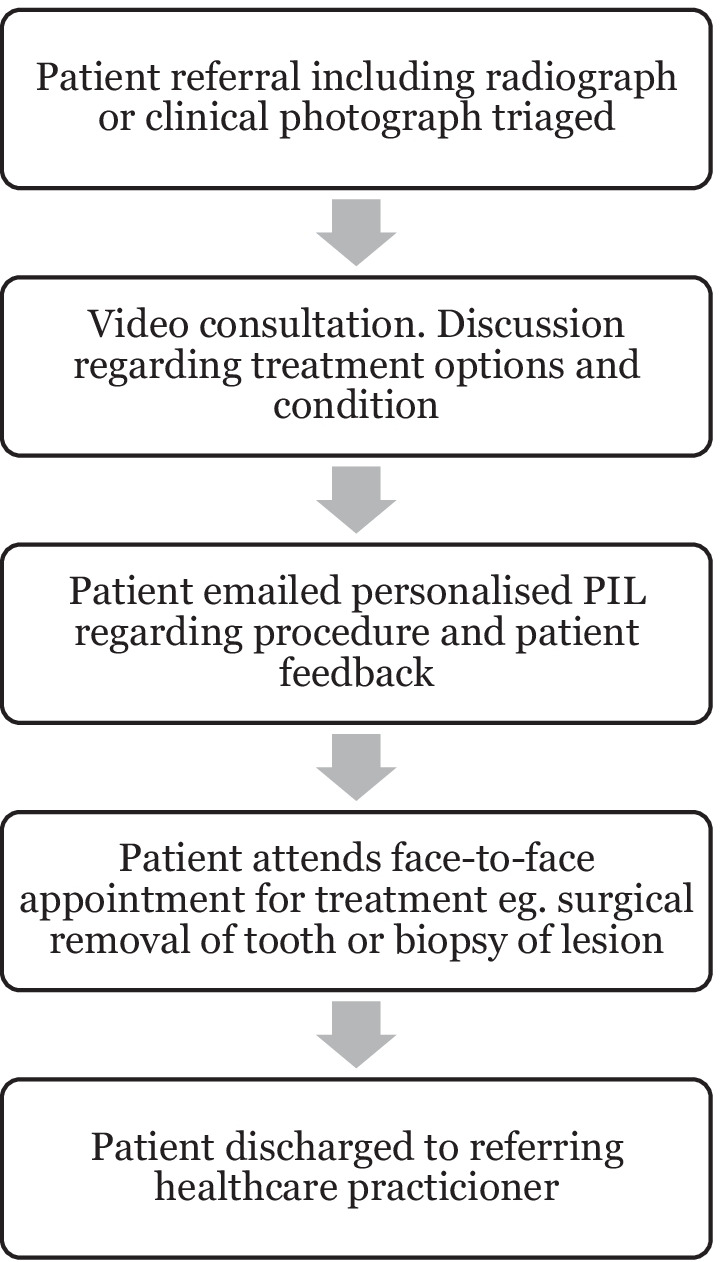
Flowchart illustrating pathway for patients seen via video consultation

The template also contained text advising patients that email correspondence with the department was not possible using this mailbox and details for routine and urgent contact were provided. Following video consultation, if a patient provided consent, they were emailed links to the information discussed in their consultation. The template allowed staff to personalise each patient email quickly by deleting the irrelevant leaflets. Digitalising leaflets and use of the clinical mailbox led to the creation of a video consultation patient pathway (Fig. 1). Virtual departmental staff training on use of the mailbox and email templates was provided with one-to-one support available where required. A standard operating procedure (SOP) was created to ensure staff practice conformed with information governance requirements. Staff were made aware that no identifiable information should be present in the emails and that it should be recorded in the patients’ notes when an email had been sent.

## Methods

**Fig. 2:**
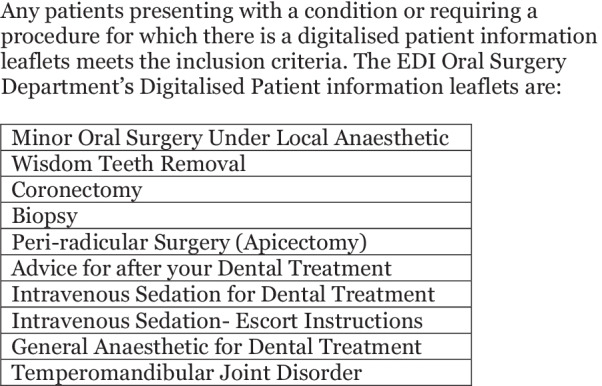
Inclusion criteria for patients requiring an information leaflet following video consultation

The current PILs used by the EDI’s Oral Surgery Department were used to create an inclusion list for patients requiring an information leaflet following video consultation (Fig. 2). Any patient presenting with a condition or requiring treatment for which there was a relevant leaflet met the inclusion criteria. This included new patients and returning patients, for example those attending for treatment planning following Cone Beam CT scans. Patients for whom there was not a relevant leaflet, for example those seen for a virtual review following surgery, did not meet the inclusion criteria. A retrospective audit was carried out with the aim to investigate if patients seen virtually received the same standard of care as patients seen face-to-face by determining departmental compliance with delivery of patient information leaflets following video consultation and to evaluate patient’s perceptions of video consultations. The clinical notes of 100 video consultations between August and September 2020 were assessed. Patients were excluded from the audit if there was no relevant patient information leaflet. Electronic clinical notes were assessed for a record of a discussion about the patient’s condition, the proposed procedure and the associated benefits and risks. If a patient information leaflet was sent following video consultation the delivery method was recorded. Anonymous patient data from the 100 virtual consultations was analysed to determine the demographic split of the patients seen. The data analysed was age, gender and The Scottish Index of Multiple Deprivation (SIMD). SIMD is a relative measure of deprivation based on postcode and is determined by seven factors: income, employment, education, health, access to services, crime and housing. SIMD quintiles rank areas from most deprived (Quintile 1) to least deprived (Quintile 5).

Following the findings of the first cycle of this audit, the department’s patient information leaflets were digitalised and a clinical mailbox introduced to allow clinicians, with consent, to email patients a hyperlink to the appropriate leaflet. A survey link was included in this email for patients to provide feedback on their video consultation and digital patient information leaflet. This survey, created using Jisc Platform, contained single choice answer questions and likert scale answer questions to assess patients’ ease of use of the virtual platform, specific technological issues and the perceived patient benefits and disadvantages to video consultations. All patients seen virtually were sent a link to the survey, regardless of if they were sent an information leaflet.

Following leaflet digitalisation and mailbox implementation a second audit cycle was completed assessing the clinical notes of 88 video consultations between February and March 2021. The same inclusion criteria was used (Fig. 2). A survey was sent to the department’s clinicians to determine perceived importance of patient information leaflets and the ease of mailbox use. Likert scale questions were used throughout and there was a free text box associated with each question to allow clinicians to provide additional comments.

## Results

### Cycle 1

Initially, 51% (n = 51) of cases met the inclusion criteria for consultations requiring a patient information leaflet. 84.3% (n = 43) of clinical notes for included cases recorded a discussion about the patient’s condition or proposed procedure and the associated benefits and risks. 15.6% (n = 8) of included patients were sent leaflets, 11.7% (n = 6) via post and 3.9% (n = 2) signposted to online information.

### Demographic split

**Fig. 3:**
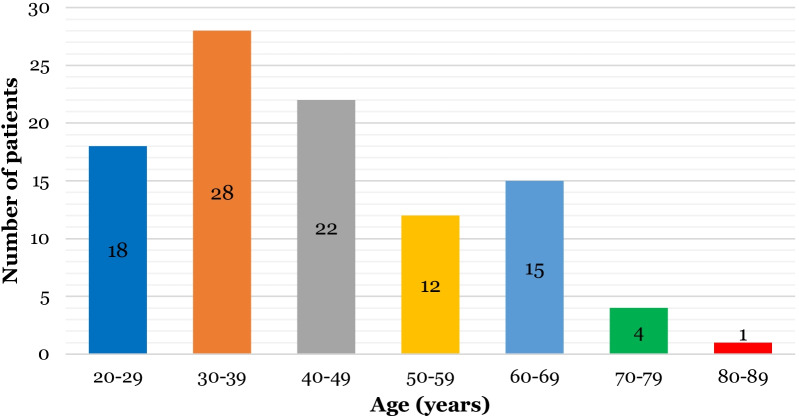
Bar chart illustrating age of patients seen for virtual consultations (n = 100)

**Fig. 4:**
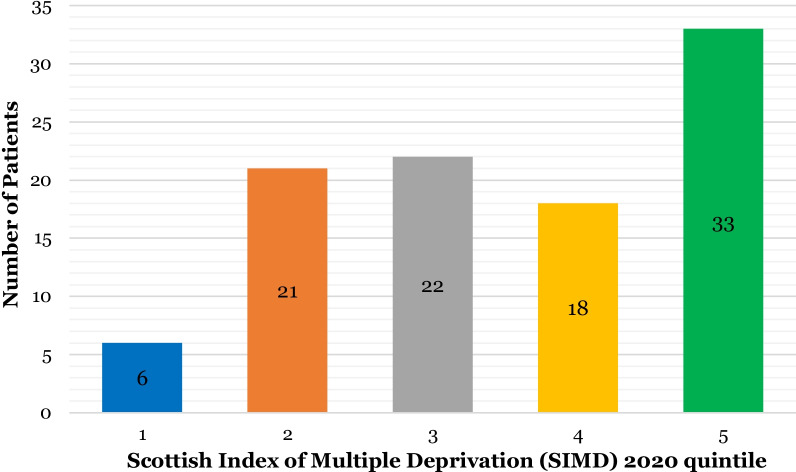
Bar chart illustrating SIMD Quintiles for patients seen for virtual consultations (n = 100)

Off the 100 patients seen, 60% (n = 60) were female and 40% (n = 40) were male. The most common age group was 30–39 years (n = 28) and the least common age group was over 80 years (n = 1) (Fig. 3). 80% (n = 80) of patients seen for virtual consultations were under the age of 60. 33% (n = 33) of patients seen were in the least deprived SIMD quintile and 6% (n = 6) were in the most deprived quintile (Fig. 4). There was a more even spread of patients in the middle quintiles, with 21% (n = 21) in quintile 2, 22% (n = 22) in quintile 2 and 18% (n = 18) in quintile 3.

### Cycle 2

Following mailbox implementation, 55% (n = 48) of cases met the inclusion criteria and 100% (n = 48) of notes for included cases had a record of discussion regarding condition, procedure, benefit and risk. 93.8% (n = 45) of patients were sent information leaflets, all via email using the clinical mailbox. These results show a significant increase in compliance of patients being sent a relevant patient information leaflet following video consultation.

### Staff feedback

**Fig. 5:**
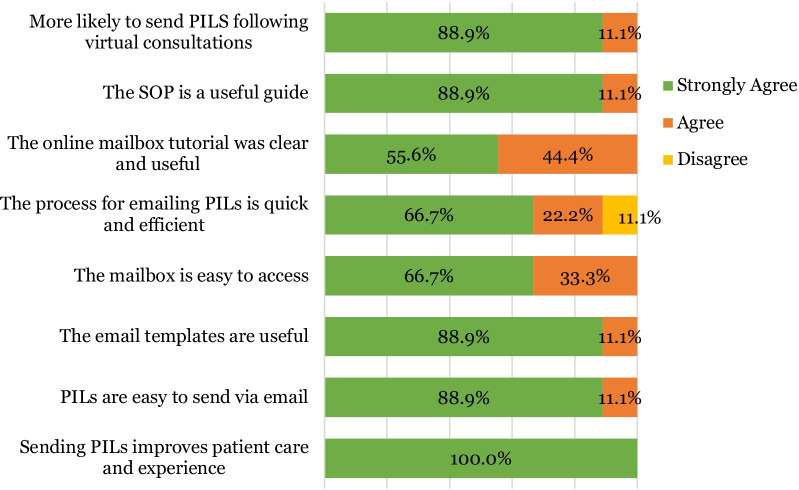
Stacked bar chart illustrating staff perceptions of using clinical mailbox to send digital PILs (n = 9)

**Fig. 6:**
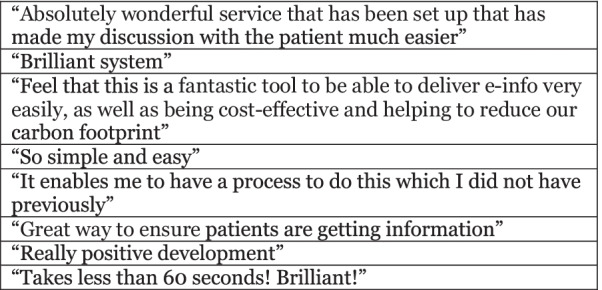
Text box with staff free text responses regarding clinical mailbox use to send digital PILs

Nine staff members of the Oral Surgery Department completed the electronic survey leading to a response rate of 69.2% (Fig. 5). 100% of respondents (n = 9) agreed that sending patient information leaflets improves patient care. 88.9% (n = 8) strongly agreed and 11.1% (n = 1) agreed that PILs were easy to send via email, the email templates were useful, the standard operating procedure was a useful guide and that they were more likely to send leaflets to patients following video consultations. All respondents agreed or strongly agreed and that the mailbox was easy to access. The majority of staff (88.9% n = 8) agreed that the process of sending leaflets was quick and efficient however one member of staff (11.1%) disagreed, noting that IT issues and slow computers delayed the process. The free text comments (Fig. 6) revealed that staff had overwhelmingly positive views about the implementation of the mailbox, remarking that it has made video consultations easier, that it is “a fantastic tool to deliver information easily” and that it has been a “really positive development”.

### Patient survey

**Fig. 7:**
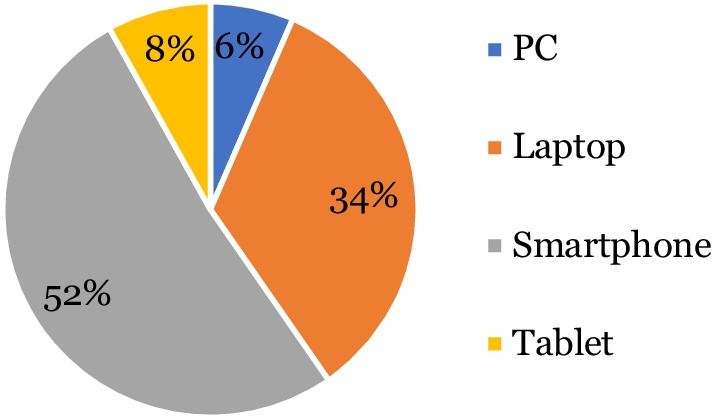
Pie chart illustrating percentage of patients using different devices to access their video consultation (n = 62)

**Fig. 8:**
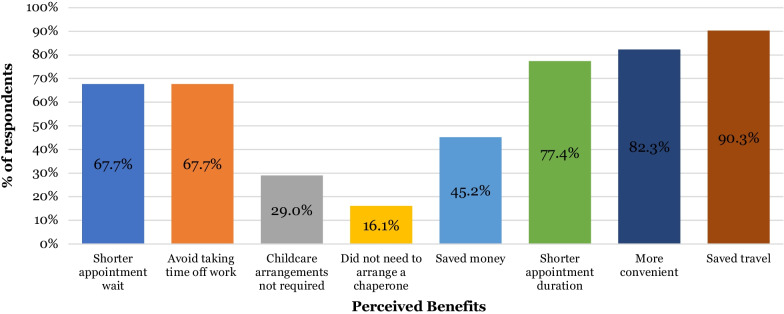
Bar chart illustrating patients’ perceived benefits of video consultations (n = 62)

All 62 patients who responded to the electronic survey were from the second cycle. Over half of patients accessed their video consultation using a smartphone (51.6% n = 32), followed by a third using a laptop (33.9% n = 21), with use of a tablet (8.1% n = 5) or a PC (6.4% n = 4) less common (Fig. 7). The majority of patients felt very comfortable (69.4% n = 43) or comfortable (21% n = 13) using technology during their appointment with only 8% (n = 5) of patients feeling very uncomfortable with this format of appointment. Most found the platform easy to use (95.2% n = 59) and the most common reported benefits (Fig. 8) were that virtual consultations avoided the need for additional travel (90.3% n = 56), were more convenient (82.3% n = 51), took less time than in person appointments (77.4% n = 48), there was a shorter wait for appointments (67.7% n = 42) and they avoided the need to take time off work (67.7% n = 42).

**Fig. 9:**
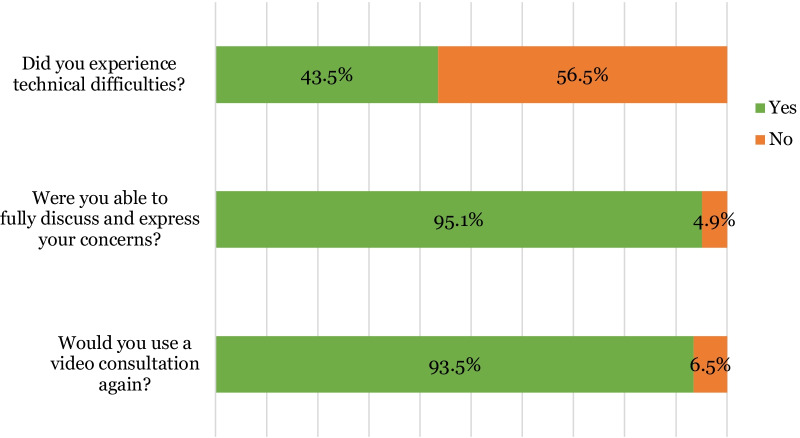
Stacked bar chart illustrating patients’ perceptions of video consultations (n = 62)

**Fig. 10:**
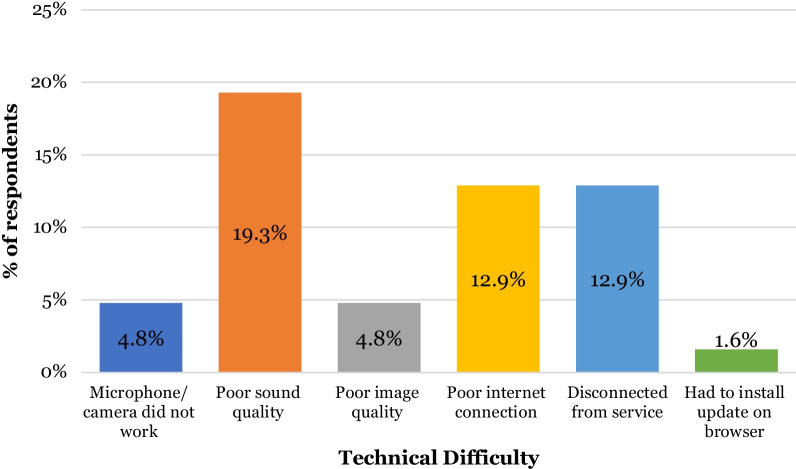
Bar chart illustrating the technical difficulties encountered by patients during video consultations (n = 62)

**Fig. 11:**
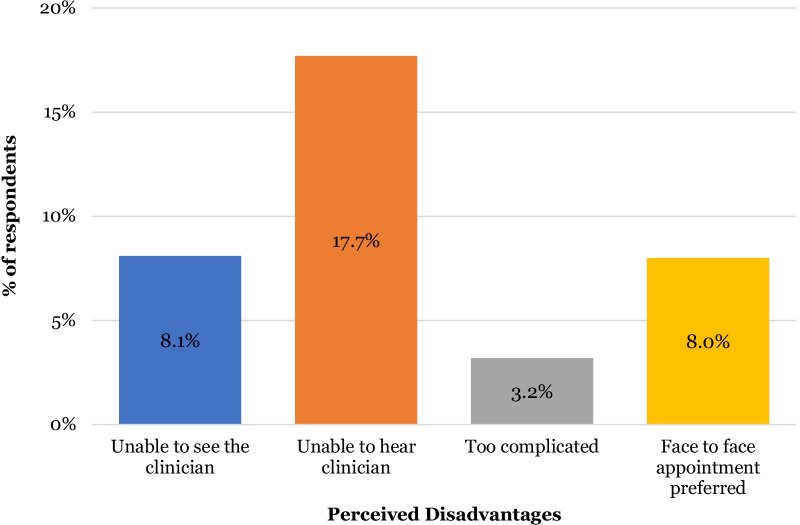
Bar chart illustrating patients’ perceived disadvantages of video consultations (n = 62)

However, 43.5% (n = 27) of patients reported encountering technical difficulties (Fig. 9). Out of the 27 reported cases of technical difficulties, the most common issues (Fig. 10) were poor sound quality (19.3%, n = 12), poor internet connection (12.9% n = 8) and disconnection from the platform (12.9% n = 8). 19 patients felt there were disadvantages to video consultations with the most common being unable to hear (17.7% n = 11) or see (8.1% n = 5) the clinician on the other side of the video. 3.2% (n = 2) felt that the platform was too complicated to use (Fig. 11).

Overall, 95.1% (n = 59) of patients felt they were able to fully discuss and express their concerns during their video consultation and 93.5% (n = 58) reported that they would be happy to use a video consultation again (Fig. 9).

**Fig. 12:**
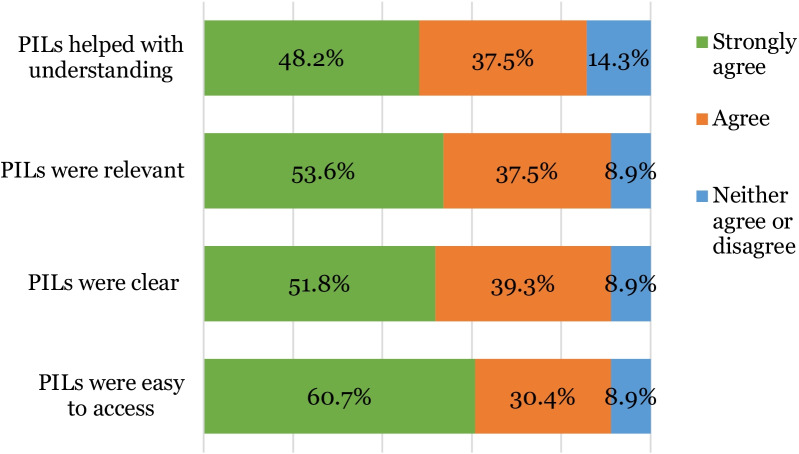
Stacked bar chart illustrating patients’ perceptions of digital PILs (n = 56)

90% (n = 56) of patients surveyed had been emailed a link to a patient information leaflet following their consultation. All leaflets were emailed to patients on the same day, the majority sent directly following consultation and the remainder sent at the end of the session. 60.7% (n = 34) of patients strongly agreed and 30.4% (n = 17) agreed that the leaflet was easy to access with 51.8% (n = 29) strongly agreeing and 39.3% (n = 22) agreeing that the leaflet was clear and easy to understand. 53.6% (n = 30) of patients strongly agreed and 37.5% (n = 21) agreed that the leaflet contained relevant information and 48.2% (n = 27) in strong agreement and 37.5% (n = 21) in agreement that the leaflet helped with understanding of a condition or proposed treatment (Fig. 12).

## Discussion

Following the Covid-19 pandemic, virtual clinics have been incorporated into practice by many dental specialities, ranging from orthodontic clinics [6] and triage for referrals [8] to head and neck oncology monitoring clinics [9]. With lengthy waiting lists following the cessation of services [15], virtual clinics can aid triage and be utilised for new patient appointments. At the Edinburgh Dental Institute Oral Surgery Department, all new patients with appropriate radiographs attached to their referral have an initial consultation virtually. These radiographs, taken by their referring practitioner, are available on patients’ electronic records. The majority of the department’s virtual consultations are for elective care. Following a discussion about treatment options and a shared decision regarding a plan, patients are then booked directly into a treatment clinic. Following their procedure, they are discharged to their referring practitioner or reviewed through a virtual clinic. If further investigations are required following the initial consultation, these are arranged and reviewed virtually. This provides an efficient patient pathway whilst reducing the need for in person appointments (Fig. 1).

The results of our study show high levels of patient satisfaction with video clinics. These results compare similarly to other recent patient satisfaction surveys on virtual dental consultations. Rahman et al. [16] reported that 91% of patients surveyed could understand the virtual system and maintain good communication and that 97% felt they could express themselves clearly as if they had an in person consultation. Reported benefits included avoidance of travel and parking. 100% agreed the virtual consultation saved time and 97% would be happy to use video for future appointments. A similar study by Parker and Chia [6] also reported high levels of patient satisfaction with 97% of patients finding their video consultation easy to use and convenient and 93.7% reporting a positive experience. 95% of patients would use a video consultation again and 90% would recommend this style of consultation to a friend. Another study by Menhadji et al. [17] similarly reported over 90% of patients felt comfortable with their video consultation, 87% found their virtual consultation useful and 79.3% would recommend video consultation to others. A literature review by Almathami et al. [18] as well as a study by Barca et al. [19] also found high levels of patient satisfaction with medical online consultations and reported virtual consultations were as good as in person consultations.

With the incorporation of video consultations into practice, it is important that patients have as similar an experience as an in person consultation. The General Dental Council state that you should “give patients the information they need, in a way they can understand, so that they can make informed decisions” and ensure patients are given “sufficient information and a reasonable amount of time to consider that information in order to make a decision” [20]. This is important for the consent process and for shared decision making regarding a patient’s treatment plan. A study by Shakir et al. [21] found that following consultation regarding third molar removal surgery, patient understanding and retention of the important information discussed was poor. Additional studies have shown that risk recall is better with written information compared to verbal information alone [22] and that verbal information should be supplemented with written or visual information [23]. Use of a clinical mailbox to send digital PILs is a time and cost-effective method of delivering pertinent information. Some virtual consultation platforms, for example, Attend Anywhere, lack a straightforward means of sharing text links with patients during consultation and there is no way of accessing the link when the consultation has ended. Use of a clinical mailbox is a flexible way of sending patient information, regardless of consultation platform or device used. Their use can also be easily incorporated into telephone clinics. Anecdotally, patients report leaflets links are easy to retrieve in their email inbox for when they wish to access the reference information later.

This project has demonstrated an increase to 94% compliance for delivering PILs following consultation. Patient feedback regarding the digital leaflets was overwhelmingly positive with over 90% agreeing they were easy to access, that the leaflet was clear and easy to read, and that it contained relevant information. 86% of patients agreed that the leaflet helped with their understanding with the remaining 14% neither agreeing nor disagreeing. A study of dermatology patients requiring surgery found that most patients preferred to receive an email with information about their surgery prior to the procedure [24]. This efficient and effective intervention allows patients to receive the same standard of care as if they were seen in person whilst ensuring they have sufficient information about their procedure.

There are many barriers to implementing change within a healthcare setting. Common reasons include: increased workload, staff commitment and attitude towards the intervention, and lack of support and training [25]. Covid-19 has pushed the use of teledentistry and telemedicine; a change that was very well received from both patients and clinicians. The clinical mailbox introduced during this project to support teledentistry was designed to be as user friendly, time-efficient and straightforward as possible to overcome these barriers. Utilising email templates containing the hyperlinks to each digital information leaflet allows clinicians to personalise each patient’s email in an efficient manner by deleting the irrelevant information. 1 h of virtual training was delivered to all department staff members with in person one-to-one sessions available as required. 3 members of staff required an additional in person session to help set up the mailbox and gain familiarity with the template system. An electronic SOP distributed to all staff contained a guide for further reference and for new members joining the department. The increase in compliance to 94% and positive staff feedback demonstrates that several barriers have been overcome to implement mailbox introduction for departmental use.

The authors are not aware of any other studies regarding dental staff perceptions of emailing patient information leaflets following virtual consultations. However, there have been several studies reporting high levels of clinician satisfaction regarding video consultations in general [17, 26, 27]. A survey of Oral and Maxillofacial consultants, who pre-pandemic held mixed views about the benefit of virtual consultations, now report more positive opinions and believe that there is a place for them in future practice [10]. The Covid-19 pandemic has forced all departments to adapt and following the positive incorporation of virtual clinics, departments appear more open to implementing new changes such as the introduction of a clinical mailbox to send PILs.

It is important to be cognisant of the limitations and barriers associated with teledentistry. Technical difficulties were encountered by 43% of our study’s respondents, with poor sound quality and poor internet connection noted as the most common. Issues with technology are cited in many studies as a barrier to successfully implementing virtual consultations [2, 8, 17, 18, 27] with one study stating that the standard of the consultation depended on the quality of devices and internet used [8]. If healthcare settings are to incorporate technology into consultations, it is crucial that there is the IT infrastructure to support this.

It is reported that 85% of British adults sent or received an email in 2020 and that for those aged over 75, internet usage has increased from 29% in 2013 to 54% in 2020 [28], highlighting the population’s increase in digital literacy and access. Therefore, moving forward there is a large cohort of patients for whom email and digital consultations may be utilised easily. Data extrapolated from the first 100 virtual consultations showed that 80% of patients seen were under the age of 60 years. One third (33%) of patients seen were ranked as living in the least deprived area of the country. Patient demographics, internet access and digital literacy are important factors to consider when assessing potential barriers to accessing virtual consultations. Six per cent of households in the United Kingdom and 18% of over 64 s lack internet access and five per cent of internet users did not feel confident using the internet [29]. Further studies into the impact of teledentistry on patient experience and level of engagement for different patient groups would be beneficial. Determining if age, gender, educational level, income and digital literacy have an effect on engagement and experience may help identify barriers to accessing care and highlight what further assistance may be required.

Concerns have also been raised regarding missed diagnoses [10], medico-legal issues [2, 9] and difficulties viewing all parts of the mouth through video [9, 27]. The majority of patients however, are still seen in person at some stage of their treatment journey with Wosik et al. [30] finding that 85% of their virtual consultations resulted in a face-to-face appointment. This allows for in person examination of the oral cavity to be completed, likely during a treatment appointment. For those seen by virtual consultation alone, a study by Perdoncini et al. [31] found that the successful diagnosis of oral lesions was comparable between virtual consultations and face-to-face consultations. Information governance is another area of concern raised however, the NHS have issued guidance to support the use of virtual consultations and email communication with patients [32, 33]. Considerations include patient consent, safeguarding confidential information, pre-agreed communications and adhering to record management policy. The email templates and SOP used in this project adhere to this guidance.

As departments move forward, implementation of a clinical mailbox can supplement virtual consultations. Virtual clinics are an excellent tool when used appropriately and they may also provide financial benefits. A previous study investigating the cost benefit analysis of treatment at EDI [34], found that a consultation appointment including a radiograph cost £250. It is estimated that the cost of a virtual appointment is £100–£150. Analysis carried out in the West Midlands NHS Trust [35] reported a reduction in failed appointments with virtual consultations and found that this consultation method was on an average of 2.5 min shorter than an in person patient appointment. This equated to an additional 52,000 h of available appointment time. Further studies are required on the cost–benefit improvement of virtual consultations on service provision [36, 37] but the cost savings for patients regarding reduced income loss, travel expenses and time have been widely reported [16, 35–37].

Amid concerns about the carbon footprint of dentistry, especially with the increased use of plastic for personal protective equipment following Covid-19 [38], digital leaflets provide an environmental and sustainable means of reducing paper waste. A bank of digital leaflets in different languages can be collated and a clinical mailbox could also be used in health promotion, for example to deliver smoking or alcohol cessation advice. Some departments have started using QR codes to allow patents to scan their leaflets [39]. It is hoped that departments may find this pathway and the use of a clinical mailbox beneficial and something to consider implementing into their practice.

## Conclusions

This project has shown both patients’ and clinicians’ positive perceptions regarding virtual clinics and how the introduction of a mailbox can enhance video consultations in an easy and efficient manner. Virtual consultations are popular amongst patients and staff, and have been shown to provide a range of benefits including increased flexibility and accessibility whilst saving time. However, departments must be cognisant of the population’s varying levels of IT literacy and access to technology. With the implementation of virtual clinics, safeguards must be in place to avoid creating additional barriers to universal access to care. Further studies into the impact of teledentistry on different demographics of the population would be beneficial to highlight groups for whom face-to-face appointments may be more appropriate and to identify what further assistance is required.

There will always be a need for in person appointments, however the implementation of virtual consultations can reduce the volume of face-to-face appointments and streamline services. Implementation of a mailbox and digital PILs, as demonstrated by this project, creates a means of delivering standardised patient information for reference, regardless of consultation type, consultation platform or patient pathway. As departments implement post-pandemic service changes, it is hoped that utilisation of a mailbox may be considered to provide multiple improvements to virtual consultations and the patient experience.

## Data Availability

The datasets used and/or analysed during the current study are available from the corresponding author on reasonable request.

## Abbreviations

EDI: Edinburgh Dental Institute
PIL: Patient information leaflet
SOP: Standard operating procedure

## References

[1] World Health Organisation (2010). Telemedicine: opportunities and developments in member states: report on the second global survey on eHealth 2009. Glob Obs eHealth Ser.

[2] Estai M, Kruger E, Tennant M, Bunt S, Kanagasingam Y (2016). Challenges in the uptake of telemedicine in dentistry. Rural Remote Health.

[3] Purohit BM, Singh A, Dwivedi A (2017). Utilization of teledentistry as a tool to screen for dental caries among 12-year-old school children in a rural region of India. J Public Health Dent.

[4] Fricton J, Chen H (2009). Using teledentistry to improve access to dental care for the underserved. Dent Clin North Am.

[5] Hurley S, Neligan M. Issue 3: Preparedness letter for primary dental care - 25 March 2020. 2020. https://www.england.nhs.uk/coronavirus/wp-content/uploads/sites/52/2020/03/issue-3-preparedness-letter-for-primary-dental-care-25-march-2020.pdf. Accessed 29 Sept 2021.

[6] Parker K, Chia M (2021). Patient and clinician satisfaction with video consultations in dentistry—part one: patient satisfaction. Br Dent J.

[7] Coulthard P, Thomson P, Dave M, Coulthard FP, Seoudi N, Hill M (2020). The COVID-19 pandemic and dentistry: the clinical, legal and economic consequences—part 1: clinical. Br Dent J.

[8] Robiony M, Bocin E, Sembronio S, Costa F, Arboit L, Tel A (2021). Working in the era of COVID-19: an organization model for maxillofacial surgery based on telemedicine and video consultation. J Craniomaxillofac Surg.

[9] da Silva HEC, Santos GNM, Leite AF, Mesquita CRM, de Souza Figueiredo PT, Dos Reis PED, Stefani CM, de Melo NS (2021). The role of teledentistry in oral cancer patients during the COVID-19 pandemic: an integrative literature review. Support Care Cancer.

[10] Al-Izzi T, Breeze J, Elledge R (2020). Following COVID-19 clinicians now overwhelmingly accept virtual clinics in Oral and Maxillofacial Surgery. Br J Oral Maxillofac Surg.

[11] Cabinet Office. Staying at home and away from others (social distancing). 2020. https://www.gov.uk/government/publications/full-guidance-on-staying-at-home-and-away-from-others/full-guidance-on-staying-at-home-and-away-from-others. Accessed 15 Oct 2021.

[12] Wallace C, Schofield C, Burbridge L, O’Donnell K (2021). Role of teledentistry in paediatric dentistry. Br Dent J.

[13] Europe Economics. Regulatory approaches to telemedicine. 2021. https://www.gmc-uk.org/about/what-we-do-and-why/data-and-research/research-and-insight-archive/regulatory-approaches-to-telemedicine. Accessed 28 Sept 2021.

[14] General Dental Council. High level principles for good practice in remote consultations and prescribing. 2020. https://www.gdc-uk.org/docs/default-source/guidance-documents/high-level-principles-remote-consultations-and-prescribing.pdf. Accessed 28 Sept 2021.

[15] NHS England. Consultant-led referral to treatment waiting times data 2021–2022. England level timeseries. 2021. https://www.england.nhs.uk/statistics/statistical-work-areas/rtt-waiting-times/rtt-data-2021-22. Accessed 3 Oct 2021

[16] Rahman N, Nathwani S, Kandiah T (2020). Teledentistry from a patient perspective during the coronavirus pandemic. Br Dent J.

[17] Menhadji P, Patel R, Asimakopoulou K, Quinn B, Khoshkhounejad G, Pasha P, Garcia Sanchez R, Ide M, Kalsi P, Nibali L (2021). Patients' and dentists' perceptions of tele-dentistry at the time of COVID-19: A questionnaire-based study. J Dent.

[18] Almathami H, Win K, Vlahu-Gjorgievska E (2020). Barriers and facilitators that influence telemedicine-based, real-time, online consultation at patients' homes: systematic literature review. J Med Internet Res.

[19] Barca I, Novembre D, Giofrè E, Caruso D, Cordaro R, Kallaverja E, Ferragina F, Cristofaro MG (2020). Telemedicine in oral and maxillo-facial surgery: an effective alternative in post COVID-19 pandemic. Int J Environ Res Public Health.

[20] General Dental Council. Standard for the Dental Team. 2013. https://standards.gdc-uk.org/Assets/pdf/Standards%20for%20the%20Dental%20Team.pdf. Accessed 8 Oct 2021

[21] Shakir D, Milori M, Ventura N, Kolokythas A (2019). What information do patients recall from the third molar surgical consultation?. Int J Oral Maxillofac Surg.

[22] Hong P, Makdessian A, Ellis D, Taylor S (2009). Informed consent in rhinoplasty: prospective randomized study of risk recall in patients who are given written disclosure of risks versus traditional oral discussion groups. J Otolaryngol Head Neck Surg.

[23] Thomson A, Cunningham S, Hunt N (2001). A comparison of information retention at an initial orthodontic consultation. Eur J Orthod.

[24] Hawkins SD, Barilla S, Williford PWM, Feldman SR, Pearce DJ (2017). Patient perceptions of text-messages, email, and video in dermatologic surgery patients. Dermatol Online J..

[25] Geerligs L, Rankin NM, Shepherd HL (2018). Hospital-based interventions: a systematic review of staff-reported barriers and facilitators to implementation processes. Implement Sci.

[26] Parker K, Chia M (2021). Patient and clinician satisfaction with video consultations in dentistry—part two: clinician satisfaction. Br Dent J.

[27] Patel T, Wong J (2020). The role of real-time interactive video consultations in dental practice during the recovery and restoration phase of the COVID-19 outbreak. Br Dent J.

[28] Office for National Statistics. Internet users, UK: 2020. 2020. https://www.ons.gov.uk/businessindustryandtrade/itandinternetindustry/bulletins/internetusers/2020. Accessed 15 Oct 2021.

[29] Ofcom. Online Nation 2021 report. 2021. https://www.ofcom.org.uk/__data/assets/pdf_file/0013/220414/online-nation-2021-report.pdf. Accessed 15 Oct 2021.

[30] Wosik J, Fudim M, Cameron B, Gellad ZF, Cho A, Phinney D, Curtis S, Roman M, Poon EG, Ferranti J, Katz JN, Tcheng J (2020). Telehealth transformation: COVID-19 and the rise of virtual care. J Am Med Inform Assoc.

[31] Perdoncini NN, Schussel JL, Amenábar JM, Torres-Pereira CC (2021). Use of smartphone video calls in the diagnosis of oral lesions: teleconsultations between a specialist and patients assisted by a general dentist. J Am Dent Assoc.

[32] NHS X. Template for email and text message communications. 2020. https://www.nhsx.nhs.uk/information-governance/guidance/template-email-and-text-message-communications. Accessed 16 Oct 2021.

[33] The Scottish Government. Covid-19 Guidance on use of email. 2020. https://www.informationgovernance.scot.nhs.uk/email-guidance. Accessed 16 Oct 2021.

[34] Pitros P, O'Connor N, Tryfonos A, Lopes V (2020). A systematic review of the complications of high-risk third molar removal and coronectomy: development of a decision tree model and preliminary health economic analysis to assist in treatment planning. Br J Oral Maxillofac Surg.

[35] The Strategy Unit NHS Midlands and Lancashire Commissioning Support Unit. The Potential Economic Impact of Virtual Outpatient Appointments in the West Midlands - A scoping study. 2018. https://www.strategyunitwm.nhs.uk/publications/potential-economic-impact-virtual-outpatientappointments-west-midlands-scoping-study. Accessed 2 Jan 2021.

[36] Greenhalgh T, Vijayaraghavan S, Wherton J, Shaw S, Byrne E, Campbell-Richards D, Bhattacharya S, Hanson P, Ramoutar S, Gutteridge C, Hodkinson I, Collard A, Morris J (2016). Virtual online consultations: advantages and limitations (VOCAL) study. BMJ Open.

[37] Daniel S, Wu L, Kumar S (2013). Teledentistry: a systematic review of clinical outcomes, utilization and costs. Am Dental Hyg Assoc.

[38] Ahmadifard A (2020). Unmasking the hidden pandemic: sustainability in the setting of the COVID-19 pandemic. Br Dent J.

[39] Sharara S, Radia S (2021). Quick response (QR) codes for patient information delivery: a digital innovation during the coronavirus pandemic. J Orthod.

